# Impact of COVID-19 during pregnancy on placental pathology, maternal and neonatal outcome – A cross-sectional study on anemic term pregnant women from a tertiary care hospital in southern India

**DOI:** 10.3389/fendo.2023.1092104

**Published:** 2023-03-21

**Authors:** M. V. Surekha, N. Suneetha, N. Balakrishna, Uday Kumar Putcha, K. Satyanarayana, J. J. Babu Geddam, Pagidoju Sreenu, B. Tulja, Raja Sriswan Mamidi, Guy A. Rutter, Gargi Meur

**Affiliations:** ^1^ Pathology and Microbiology Division, Indian Council of Medical Research-National Institute of Nutrition, Hyderabad, India; ^2^ Obstetrics & Gynecology Department, Government Area Hospital, Nampally, Hyderabad, India; ^3^ Department of Statistics, Apollo Hospitals Educational and Research Foundation (AHERF), Hyderabad, India; ^4^ Clinical Epidemiology Division, Indian Council of Medical Research-National Institute of Nutrition, Hyderabad, India; ^5^ Clinical Division, Indian Council of Medical Research-National Institute of Nutrition, Hyderabad, India; ^6^ Centre of Research of Centre Hospitalier de l'Université de Montréal (CRCHUM), Faculty of Medicine, University of Montreal, Montreal, QC, Canada; ^7^ Section of Cell Biology and Functional Genomics, Imperial College London, London, United Kingdom; ^8^ Lee Kong Chian School of Medicine, Nanyang Technological University, Singapore, Singapore; ^9^ Cell Biology Division, Indian Council of Medical Research-National Institute of Nutrition, Hyderabad, India

**Keywords:** COVID-19, SARS-CoV-2, pregnancy, cord blood, placenta, anemia

## Abstract

**Background:**

SARS-CoV-2 infection during pregnancy may cause adverse maternal, neonatal and placental outcomes. While tissue hypoxia is often reported in COVID-19 patients, pregnant women with anemia are suspected to be more prone to placental hypoxia-related injuries.

**Methods:**

This hospital-based cross-sectional study was conducted between August-November 2021, during COVID-19 second wave in India. Term pregnant women (N=212) admitted to hospital for delivery were enrolled consecutively. Since hospital admission mandated negative RT-PCR test for SARS-CoV-2 virus, none had active infection. Data on socio-demography, COVID-19 history, maternal, obstetric, and neonatal outcomes were recorded. Pre-delivery maternal and post-delivery cord blood samples were tested for hematological parameters and SARS-CoV-2 IgG. Placentae were studied for histology.

**Results:**

Of 212 women, 122 (58%) were seropositive for SARS-CoV-2 IgG, but none reported COVID-19 history; 134 (63.2%) were anemic. In seropositive women, hemoglobin (*p*=0.04), total WBC (*p*=0.009), lymphocytes (*p*=0.005) and neutrophils (*p*=0.02) were significantly higher, while ferritin was high, but not significant and neutrophils to lymphocytes (*p*=0.12) and platelets to lymphocytes ratios (*p*=0.03) were lower. Neonatal outcomes were similar. All RBC parameters and serum ferritin were significantly lower in anemic mothers but not in cord blood, except RDW that was significantly higher in both, maternal (*p*=0.007) and cord (*p*=0.008) blood from seropositive anemic group compared to other groups. Placental histology showed significant increase in villous hypervascularity (*p*=0.000), dilated villous capillaries (*p*=0.000), and syncytiotrophoblasts (*p*=0.02) in seropositive group, typically suggesting placental hypoxia. Maternal anemia was not associated with any histological parameters. Univariate and multivariate logistic regression analyses of placental histopathological adverse outcomes showed strong association with SARS-CoV-2 seropositivity but not with maternal anemia. When adjusted for several covariates, including anemia, SARS-CoV-2 seropositivity emerged as independent risk factor for severe chorangiosis (AOR 8.74, 95% CI 3.51-21.76, *p*<0.000), dilated blood vessels (AOR 12.74, 95% CI 5.46-29.75, *p*<0.000), syncytiotrophoblasts (AOR 2.86, 95% CI 1.36-5.99, *p*=0.005) and villus agglutination (AOR 9.27, 95% CI 3.68-23.32, *p*<0.000).

**Conclusion:**

Asymptomatic COVID-19 during pregnancy seemed to be associated with various abnormal placental histopathologic changes related to placental hypoxia independent of maternal anemia status. Our data supports an independent role of SARS-CoV-2 in causing placental hypoxia in pregnant women.

## 1 Introduction

Coronavirus disease -2019 (COVID-19) pandemic has recently affected the whole world. The first case was reported from Wuhan (China) in the year 2019 (1) and India reported its first case on 30th January 2020 (2). Pregnant women are among the more vulnerable groups to be adversely affected by COVID-19 due to their physiological immunodeficiency (3, 4). While many studies have attempted to evaluate the effects of COVID-19 on the common population (5, 6), but till date only a limited number (7–9) focused on pregnant women. COVID-19 infection was reported to cause increase in preterm birth rates, varying from 5 to 41% (7–9)in different study population. Preterm rupture of membranes (PROM) was another frequent finding in COVID-19 during pregnancy with rates being 6.1, 20.7 and 26.5% in three different studies (8–10). A retrospective observational study from India found higher incidence of severe oligohydramnios and cesarean section during the second wave, while high frequency of preterm deliveries (24-27%) and low birth weight during both waves (11), which was also observed by others in maternal COVID-19 (12). Neonatal deaths or stillbirths were found occasionally, especially in cases with severe or critical disease (13–15), while intrauterine growth retardations were also frequently reported (16). However, majority of the studies reporting on neonatal outcomes found no serious adverse outcomes in neonates born to SARS-CoV-2-positive mothers (17–19).

Entry of SARS-CoV-2 virus into the cells trigger cascades of immune responses, prompting production of pro-inflammatory cytokines (Interferon 1/IFN1, Tumor necrosis factor-alpha/TNF-α, Interleukins/IL-16,33,25) and activation of CD4^+^ and CD8^+^ T lymphocytes (20). One study also reported presentation of anemia with decreased hemoglobin (Hb) levels and pathologically increased ferritin levels in COVID-19 in their subjects (21). Anemia affects about 50% of pregnant women in developing nations and is an indirect major cause of mortality in mothers (22). Prevalence of anemia in pregnant women in India was reported to be 52.2%, and 53.2% in Telangana state as per the latest National Family Health Survey-5 (2019-21) (23). Despite a high burden of anemia in pregnant women, there were almost no studies reporting on the effects of COVID-19 in pregnant women, fetus and newborns, as pre-existing anemia could be an additional complicating factor. Tissue hypoxia is one of the common adverse effects of COVID-19 being reported, which is caused by a hypercoagulable state. Anemia is presumed to be another potential independent risk factor for tissue hypoxia pertaining to lower hemoglobin and lower physiological capacity for oxygen transport, when faced with increased demand, such as placental tissue.

The placenta forms an important mediator for the transfer of nutrients and oxygen from mother to fetus. Diverse maternal conditions, leading to morphological changes in the organ can influence the placenta’s functions (24). Maternal SARS-CoV-2 infection and its effect on the placenta and the fetus has been a major concern. A few studies investigated placental histopathology in COVID-19 patients, where histomorphological and ultrastructural changes were reported (25–27), but none of these studies could come up with specific features or hallmark changes in the placenta. Further, impact of maternal COVID-19 on pregnancy and neonatal outcomes in a population with high anemia burden remains largely unknown.

We undertook this study during the waning phase of the second wave of COVID-19 in India to understand if asymptomatic/mild COVID-19 during pregnancy was associated with any adverse maternal and fetal outcomes, as we suspected that SARS-CoV-2 infection might seriously compromise the peripheral oxygen supply to placenta and induce tissue hypoxia.

## 2 Materials and methods

### 2.1 Study design and recruitment of subjects

This cross-sectional study was conducted in a government maternity hospital in Hyderabad city of Telangana state. Consecutive pregnant women (N=212) in labor, admitted for delivery in the hospital, and who were willing to participate were enrolled after taking written informed consent. As per the COVID-19 protocol followed by the hospital during the pandemic, all women were required to undergo compulsory RT-PCR screening test for SARS-CoV-2 viral RNA at the time of admission and were admitted onlyif tested negative. Hence, all our participants, by default, were negative for any active infection during delivery.None had received any COVID-19 vaccine, as the immunization program for pregnant women did not start during the study period. Women were enrolled between August 2021 and November 2021, and only in the first half (till 2 pm) of the weekdays. The study was carried out according to ‘The Code of Ethics of the World Medical Association (Declaration of Helsinki)’ after obtaining approval from Institutional Ethical Committee (IEC) of ICMR-NIN as well as the maternity hospital.

Sample size was calculated by assuming the prevalence of SARS-CoV-2 in pregnancies as 15%, with a 95% confidence level, 80% power, which was 195. All women aged between 18–49 years, at 37-40 weeks of gestation, who were either primi or multiparous, and who did not receive any treatment for viral infections in their last trimester of pregnancy, were allowed to take part in the study. Those suffering from kidney disease, rheumatoid disease, diabetes mellitus, hypertension, acquired immune deficiency syndrome (AIDS), on immunosuppressant drugs, and who had complicated conditions such as ectopic pregnancy and hydatidiform mole were not included in the study.

### 2.2 Data collection

Complete epidemiologic history, clinical signs, symptoms, obstetric and immunization history, and outcome data of the participants were collected through a structured pre-tested questionnaire (**Supplementary-Annexure I**). The information on fever, cough, cold and other symptoms of COVID-19 were self-reported by the subject. Gestational age was calculated from the first reported day of last menstrual period (LMP). A digital balance (SECA scale, SECA robusta 813, Hamburg, Germany) was used to record the weight of the mothers to the nearest 100 g. Height was measured using a stadiometer to the nearest 0.1 cm (SECA 213 portable stadiometer). Maternal body mass index (BMI) was calculated using weight and height at baseline (kg/m^2^). Newborns were weighed without diapers and their crown-heel length was measured to the nearest 0.1cm using an infantometer (SECA). APGAR (appearance, pulse, grimace, activity, and respiration) scores at 1 min and 5 min were recorded by the attending neonatologist.

### 2.3 Blood sample collection and processing

Venous blood sample (5 ml) was drawn from all the subjects by venipuncture from the antecubital vein before delivery under strict aseptic conditions. After delivery, 5 ml of cord blood was collected by trained study personnel in the labor room in vacutainers (Beckton Dickinson, USA) with ethylene diaminetetraacetate (EDTA).For hematological parameters,whole blood was analyzed within six hours of collection in an automated hematoanalyzer (ADVIA 120, Seimens, Germany) for hemoglobin (Hb), packed cell volume (PCV), red blood cell count (RBC), mean corpuscular volume (MCV), mean corpuscular hemoglobin (MCH), mean corpuscular hemoglobin concentration (MCHC), red cell distribution width coefficient of variation percentage (RDW-CV%), platelets distribution width (PDW) and mean platelet volume (MPV).The serum from both maternal and cord blood was separated by centrifugation for 15 minutes at 1000 x *g* at room temperature and aliquots were stored at -20°C till further analysis.Anti-SARS-CoV-2 IgG was estimated in all serum samples by Enzyme-linked Immunosorbent Assay (ELISA) using Covid Kavach ELISA kit (ICMR-NIV, Pune and Zydus Diagnostics, Ahmedabad, India) following manufacturer’s instructions (**Supplementary- Annexure II**) using an ELISA reader (Model No: BioTek Synergy HT, USA).Serum ferritin was measured in all samples by ELISA (Calbiotech, El Cajon, CA, USA).

### 2.4 Placental analysis

The placentae after delivery were immediately placed in adequate volume of 10% neutral buffered formalin and brought for grossing. The placentas were fixed for 72 hours, and examined for gross abnormalities and measurements. They were cut at 1cm intervals and into 3 mm thick tissue sections. Three sections from the placenta parenchyma, one section from membranes and two cross sections of cord were studied from each placenta. After overnight processing of the tissues in a tissue processor (Automated Vacuum tissue processor, Shadon Excelsior ES, by Thermo Scientific Fisher, Ramsey, USA) these sections were embedded in paraffin (Histocentre 3, Shandon, Thermo electron corporation, Fischer Scientific, Singapore) and tissue blocks were prepared. Each block was cut to 4-5µm thick sections on a microtome (Leica RM 2155,Nussloch, Germany) and stained (Sakura Tissue Tek DRS Autostainer, Finetek, Europe) with hematoxylin and eosin stain (H&E), mounted on glass slides and examined under Nikon Eclipse E800 microscope (Nikon Instruments, Florida, USA). Histopathological analysis was done according to the Amsterdam protocol (28). The H&E stained sections were studied for histopathological findings like inflammation, vascularity, infarcts, calcification, vasculitis etc. and were counted for statistical analysis. Chorangiosis was diagnosed based on the criteria laid down by Altshuler (29). Grading of amniochorionitis was done as follows: Grade1- focal areas of neutrophils infiltration, Grade 2- neutrophils infiltration in 50% of the section, Grade 3-neutrophils infiltrating >80% of the section. Vascularity of villi was graded as normal chorionic villi that contain <5 capillaries in 10 high-power microscopic fields, and larger numbers are defined as hypervascularity; Grade 1 : 5 to 7 capillaries per villi, Grade 2 : 7 to 10 capillaries per villi, and Grade 3 : hypervascularity also known as chorangiosis is characterized by >10 capillaries in more than 10 terminal chorionic villi in several areas of the placenta. Dilated blood vessels were graded as follows: Grade 1- mildly dilated and Grade 2- moderate to severely dilated blood vessels with large lumina occupying more than 80% of the villi. Number of syncytiotrophoblasts were graded as, Grade 1 - normal with few scattered syncytiotrophoblasts, Grade 2 - increased number with the formation of syncytial knots. Fibrin deposition was graded as, Grade 1 - 10-20% of the section, Grade 2 - 20-50% of the section, Grade 3 - >50% of the section.

### 2.5 Statistical analyses

IBM SPSS (version 24.0, SPSS Inc. IBM, USA) was used for all statistical analysis. Mean and standard deviation (SD) were calculated for all continuous variables and proportions were calculated for qualitative variables. Mean values of blood and histopathological variables of mothers were compared based on their SARS-CoV-2 antisera positivity and hemoglobin (Hb) status using *t *test or Mann Whitney U test, whenever assumptions of equality of variances and normality were violated. Chi-square test was performed to study the association between categorical variables and outcomes. Spearman rank correlation coefficients were calculated to study the relationships between maternal and cord blood parameters including SARS-CoV-2 IgG. ANOVA was done for comparing multiple groups with or without COVID-19 and anemia. Univariate and multivariate logistic regression were done and models were built to identify risk factors for placental deformities. Odds ratio (OR) were calculated for potential independent variables including SARS-CoV-2 infection and anemia, and other covariates for different placental histopathologic outcomes as dependent variable. Results were considered statistically significant when *p* value was <0.05.

## 3 Results

### 3.1 Sociodemographic and maternal variables and neonatal outcome among SARS-CoV-2 antibody seropositive and seronegative groups

Of the total 212 term pregnant women enrolled during the study period, 122 (58%) were positive for SARS-CoV-2 antisera, while 90 (42%) were negative. Through self-reporting by the subjects, it was found that none had developed any symptom of COVID-19, such as cough, cold, or fever during their entire gestational period to indicate if they were infected. Hence, we concluded that those who developed SARS-CoV-2 antisera had asymptomatic COVID-19. Of the 212, 63.2% (n=134) women were anemic. As shown in **Table 1**, comparison of seropositive and negative women found that they were of almost comparable age (24.11 vs. 23.58 yr), mean body weight (62.66 vs 60.98 kg) and BMI (26.45 vs 24.72 kg/m^2^), but more women in positive group were in overweight category (65.5 vs. 76.7%). While maternal education level was comparable between the groups, a significant difference was noted in monthly family income, being lower in the positive group (*p*=0.04), and more women belonged to the community of other backward class (OBC) (*p*=0.06). About 92% (n=194 of 210) women had >=4 antenatal checkups, >94% (n=199 of 210) had two Tetanus Toxoid injections, and >98% (n=207 of 210) had taken 100 tablets of IFA (Iron and folic acid supplementation) supplementation during their pregnancy course(dose: 100 mg Fe and 500 µg folic acid) and there was no difference between the seropositive and negative groups. The mean gestational age between the two groups was similar (38.22 vs 38.73 weeks).

**Table 1:** Socio-demography, maternal features and neonatal birth outcomes of the study participants based on SARS-CoV-2 serological status.

Maternal characteristics (N=210)		SARS-CoV-2 serological status		*p*
		Positive (n=122)	Negative (n=88^#^)	
Maternal age (yr)	Mean (SD)	24.11 (3.65)	23.58 (3.19)	0.25
Weight (kg)	Mean (SD)	62.66 (9.89)	60.98 (10.47)	0.23
BMI category	<18.5, n (%)	1 (0.9)	4 (4.6)	0.29
	18.5-23, n (%)	26 (22.4)	26 (29.9)	
	>=23, n (%)	89 (76.7)	57 (65.5)	
Educational status	No school, n (%)	16 (13.1)	15 (16.7)	0.69
	Up to secondary, n (%)	97 (79.5)	70 (77.8)	
	Degree, n (%)	9 (7.4)	5 (5.6)	
Occupation	Working, n (%)	4 (3.3)	2 (2.2)	0.63
	Housemaker, n (%)	117 (95.9)	85 (95.5)	
Family monthly income (INR^$^)	<5000, n (%)	40 (32.8)	16 (17.8)	**0.04**
	5000-10,000, n (%)	64 (52.5)	61 (67.8)	
	10,000-50,000, n (%)	18 (14.8)	12 (13.3)	
	>50,000, n (%)	0	1 (1.1)	
Community	SC, n (%)	1 (0.8)	4 (4.4)	0.06
	ST, n (%)	6 (4.9)	5 (5.6)	
	OBC, n (%)	85 (69.7)	60 (66.7)	
	OC, n (%)	18 (14.8)	19 (21.1)	
	Others, n (%)	12 (9.8)	2 (2.2)	
Placental weight (g)	n (mean ± SD)	122 (410.84 ± 84.31)	90 (420.60 ± 78.49)	0.39
Neonatal outcome^$^				
Newborn weight (kg)	n (mean ± SD)	100 (2.85 ± 0.47)	76 (2.87± 0.43)	0.82
Newborn length (cm)	n (mean ± SD)	97 (48.65 ± 2.65)	68 (49.77 ± 2.40)	**0.006**
Gestation age (weeks)	n (mean ± SD)	102 (38.22 ± 1.05)	78 (38.73 ± 5.82)	0.38
APGAR score 1 min	n (mean ± SD)	122 (6.20 ± 3.01)	92 (6.21± 3.11)	0.99
APGAR score 5 min	n (mean ± SD)	100 (9.43 ± 0.68)	74 (9.51± 0.58)	0.39

For quantitative variables, comparison of mean for normally distributed dataset was done by parametric t test, and otherwise non-parametric Mann-Whitney U test. For categorical variables, Chi-square test was done.^#^Maternal variables in were missing for 2 cases and mean was calculated of n=210, unless specified otherwise. ^$^For some cases, few parameters were missing in data records, due to staff shortage at labor room on certain days. SD, standard deviation; SC, scheduled caste; ST, scheduled tribe; OBC, other backward class; OC, open category; APGAR, Appearance, Pulse, Grimace, Activity, and Respiration; UC, umbilical cord; INR- Indian Rupee. ^$^1 USD ~ 75 INR as in the August-Nov, 2021. Values in bold signifies p<0.05.

None of the 212 participants had weakness, dyspnoea, palpitation, or chest pain, and none showed signs of koilonychia and jaundice, but two women had developed pedal edema and three had pallor. Among obstetric outcomes, of the 212 pregnancies, there were cases of oligohydramnios (n=51), post-dated pregnancy (n=12), gestational hypertension (n=2), cephalopelvic disproportion (n=12), premature rupture of membranes (PROM) (n=22), placenta previa (n=1), Rh-ve pregnancy (n=8), meconium-stained liquor (MSL) (n=5) and pre-eclampsia (n=3). There were 3 cases of still births and 7 cases of spontaneous abortions.

Among the neonatal outcomes (**Table 1**), birth weight (2.85 vs. 2.87 g), APGAR scores at 1 min (6.20 vs. 6.21) and 5 min (9.43 vs. 9.5), gestational age were not different for newborns of seropositive and negative mothers. However, newborn length was lesser in seropositive group when compared to the negative group and the difference was significant (*p*=0.006).

### 3.2 Hematological variables based on SARS-CoV-2 antibody in maternal and cord blood

Hematological parameters in SARS-CoV-2 antibody seropositive and negative mothers and cord blood specimens were compared (**Table 2**). Maternal hemoglobin (*p*=0.04), WBC (*p*=0.009), MCHC (*p*=0.04), absolute lymphocyte (*p*=0.005), and absolute neutrophils (*p*=0.02) were significantly higher in seropositive mothers while, NLR (*p*=0.12) and PLR (*p*=0.03) were lower in seropositive mothers. Unlike maternal hematology parameters, only RDW (*p*=0.02) and PDW (*p*=0.005) were significantly different between the two groups in cord blood, while remaining parameters, although showed some difference, but did not vary significantly. Serum ferritin level was similar between seropositive and negative samples for both maternal and cord blood. While SARS-CoV-2 IgG level correlated well between maternal and cord blood samples (**Figure S1**), there were few exceptional cases where antibody was detected either in mother or in cord blood. Spearman correlations (**Table S1**) performed between maternal and cord blood that showed significant positive correlation for presence of IgG antibodies (**Figure S1**) in SARS-CoV-2 seropositive mother’s blood and seropositive cord blood (r=0.42, 95% CI 0.26-0.56, 2-tailed *p*<0.0001) and for monocytes % (r=0.37, *p*<0.001), but not for Hb (r=0.15, *p* =0.12), WBCs (r=0.06, *p*=0.51), absolute lymphocyte count (r=0.07, *p*=0.46) and absolute monocyte count (r=0.10, *p*=0.29). Other parameters including absolute neutrophil count (r = -0.04, *p*=0.69), NLR (r=-0.06, *p*=0.50) and PLR (r=-0.18, *p*=0.05) showed negative correlations between SARS-CoV-2 antisera positive mother and COVID-19 antisera positive cord blood but the correlation was significant only for PLR.

**Table 2:** Comparison of haematological parameters in SARS-CoV-2 antisera positive and negative maternal and cord blood samples.

^#^Parameters	Maternal blood			Cord blood		
	Positive (n=122)	Negative (n=90)	*p*	Positive (n=134)	Negative (n=65)	*p*
	Mean (SD)	Mean (SD)		Mean (SD)	Mean (SD)	
Hemoglobin (g/dl)	10.26 (1.91)	9.64 (2.48)	**0.04**	11.87 (2.36)	11.92 (2.38)	0.87
Ferritin (ng/ml)	41.6 (62.52)	44.0 (65.42)	0.253	107.88 (78.57)	124.23 (93.49)	0.11
WBC (10^3^/µl)	11.01 (3.29)	9.83 (3.23)	**0.009**	9.33 (3.86)	10.01 (11.41)	0.55
RBC (10^6^/µl)	4.03 (0.64)	3.87 (0.86)	0.12	3.62 (0.68)	3.62 (0.67)	0.99
PCV (%)	32.62 (5.29)	30.92 (7.25)	0.06	36.88 (6.80)	37.19 (6.97)	0.75
MCV (fl)	81.15 (8.78)	79.78 (8.21)	0.25	102.11 (7.33)	102.99 (5.46)	0.35
MCH (pg)	25.52 (3.67)	24.87 (3.43)	0.19	32.86 (2.97)	33.00 (2.27)	0.73
MCHC (g/dl)	31.35 (1.69)	30.87 (1.81)	**0.04**	33.55 (14.98)	32.045 (1.23)	0.37
Plt (10^3^/µl)	252.42 (84.5)	232.5 (80.20)	0.08	138.02 (95.62)	124.47 (93.65)	0.32
Lymph.absol	2.44 (0.90)	2.07 (0.96)	**0.005**	3.90 (2.36)	3.48 (2.08)	0.18
Lymphocyte (%)	23.02 (8.03)	22.37 (11.83)	0.63	40.49 (15.36)	39.72 (18.05)	0.74
Mono.absol.	0.35 (0.26)	0.30 (0.15)	0.09	0.91 (1.78)	0.69 (0.28)	0.19
Monocyte (%)	3.22 (3.24)	3.45 (2.60)	0.59	7.94 (4.15)	8.21 (2.97)	0.62
Neutro.absol.	8.25 (3.01)	7.35 (2.55)	**0.02**	4.44 (2.15)	4.32 (2.38)	0.70
Neutrophil (%)	72.39 (13.55)	73.85 (13.71)	0.44	48.54 (13.77)	47.08 (13.62)	0.46
NLR	3.69 (1.75)	4.07 (1.78)	0.12	1.37 (1.19)	1.19 (0.67)	0.22
PLR	109.87 (40.80)	124.56 (54.86)	**0.03**	37.97 (27.95)	33.43 (25.43)	0.25
RDW (%)	15.64 (3.04)	15.63 (3.74)	0.98	15.23 (1.74)	14.73 (0.93)	**0.02**
MPV (fl)	6.81 (1.07)	6.82 (1.10)	0.95	7.49 (1.40)	7.45 (1.30)	0.85
PDW (%)	16.90 (1.05)	17.07 (0.92)	0.24	18.38 (0.98)	18.85 (1.19)	**0.005**

#The mean values were compared between SARS-CoV-2 seropositive and negative subjects by parametric t test for normally distributed data set, or otherwise non-parametric Mann-Whitney U test was done. A p value <0.05 was considered significant and given in bold. BMI, body mass index; WBC, white blood cells count; RBC, red blood cells count; MCV, mean corpuscular volume; MCH, mean corpuscular haemoglobin; MCHC, mean corpuscular hemoglobin concentration; PCV, packed cell volume; RDW, red cell distribution width, Plt, platelets; MPV, mean platelets volume; PDW, platelet distribution width; Lymp.absol, absolute lymphocyte count; Mono.absol, absolute monocyte count, Neutro.absol, absolute neutrophil count; NLR, neutrophils to lymphocyte ratio; PLR, platelets to lymphocytes ratio; IgG, Immunoglobulin; fl, femtoliter; dl, decilitre; pg, picogram.

### 3.3 Hematological and other variables based on SARS-CoV-2 antisera and maternal anemia

We compared hematological parameters of maternal and cord bloodsamples between SARS-CoV-2 antibody positive and negative groups, based on maternal anemia status and shown only the parameters that were significantly different by one-way ANOVA (**Table 3**). We found, among the seropositive group, there was a difference of about 2.5 g/dl in hemoglobin between anemic and non-anemic group, while in seronegative group the difference was about 3.5 g/dl. *Post-hoc* multiple comparison between and within the groups for hemoglobin showed that mean hemoglobin level was significantly different between the anemic and normal subjects in our cohort. We observed that among the non-anemic subjects, COVID-19 did not alter hemoglobin level. However, within the anemic subjects, hemoglobin level was significantly different (~0.6 g/dl) between seropositive and negative groups. Serum ferritin level was significantly lower in seronegative anemic women when compared to seronegative non-anemic group (*p*=0.004), however, serum ferritin level was found to be raised in the anemic seropositive group (43.9 vs. 27.8 ng/ml), maybe due to infection, when compared to the seronegative anemic group (**Table 3**). In seropositive mothers with anemia, all the red cell parameters, including hemoglobin, RBC, PCV, MCV, MCH, and MCHC were significantly lower than in non-anemic mothers (**Table 3**). Among other parameters WBC, neutrophils, lymphocytes were significantly lower while PLR and RDW were higher in anemic mothers. Maternal variables such as age, height, weight, and BMI were not significantly different between and within groups. Obstetric variables, such as systolic and diastolic blood pressure, oxygen saturation % (SpO2), pulse rate, respiratory rate and heart rate were analyzed and found to be similar among the groups. Cord blood examination showed, except for RDW and PDW, almost similar hematological features between the groups, and while most of the red cell and white blood cell parameters were lower in anemic conditions, but the difference was not significant. RDW percentage, which describes the variability in red cell size, were generally higher in the anemic mothers, irrespective of SARS-CoV-2 serological status, however, in the cord blood, we noticed significantly higher variation in red cell size in anemic and seropositive group compared to others. There was no difference in cord blood hemoglobin, WBC, RBC, absolute lymphocytes, neutrophils, monocytes, and other hematological parameters. We failed to notice any difference in newborn parameters, such as weight, gestational age, APGAR scores at 1 min and 5 min, except neonatal length, which was slightly lower for those born to anemic and seropositive mothers compared newborns of other groups. Further *post hoc* multiple comparison, showed significant difference of -1.642 cm between the seropositive and negative groups within anemic mothers, but none within non-anemic mothers.

**Table 3:** Comparison of haematological parameters in SARS-CoV-2 antisera positive and negative mothers and cord blood samples based on anaemia status of the mother.

Maternal blood (N=212)					
Parameters	SARS-CoV-2 antisera positive		SARS-CoV-2 antisera negative		*p*
	Anaemic (n=72)	Normal (n=50)	Anaemic (n=62)	Normal (n=28)	
Haemoglobin (g/dl)	9.17 (1.73)^a,b,c^	11.83 (0.64)^a,d^	8.57 (2.22)^b,d,e^	12.02 (0.79)^c,e^	**0.000**
Ferritin (ng/ml)	43.9 (80.27)	44.2 (38.13)	27.8 (44.85)^a^	71.2 (84.26)^a^	**0.000**
WBC (10^3^/µl)	10.57 (3.31)^a^	11.66 (3.18)^b^	9.10 (2.92)^a,b,c^	11.43 (3.33)^c^	**0.000**
RBC (10^6^/µl)	3.90 (0.75)^a,b,^	4.23 (0.36)^a,d^	3.67 (0.94)^d,e^	4.31 (0.37)^b,e^	**0.000**
PCV (%)	30.05 (5.37)	36.32 (1.91)	28.11 (6.97)	37.16 (2.24)	**0.000**
MCV (fl)	77.60 (8.86)	86.26 (5.61)	76.74 (7.39)	86.50 (5.51)	**0.000**
MCH (pg)	23.71 (3.30)	28.14 (2.37)	23.46 (2.96)	27.99 (2.11)	**0.000**
MCHC (g/dl)	30.49 (1.56)	32.58 (0.93)	30.36 (1.31)	31.99 (2.25)	**0.000**
Lymph.absol	2.44 (1.05)	2.44 (0.65)	1.89 (0.84)	2.47 (1.09)	**0.002**
Neutro.absol	7.8 (2.9)	8.87 (3.14)	6.91 (2.38)	8.31 (2.67)	**0.003**
PLR	114.19 (46.47)^a^	103.65 (30.21)	133.54 (58.89)	104.69 (38.62)	**0.003**
NLR	3.57 (1.75)^a^	3.85 (1.76)	4.19 (1.82)	3.81 (1.69)	0.255
RDW (%)	16.26 (2.95)^a,b^	14.77 (2.98)^a,c^	16.22 (4.20)^c,e^	14.34 (1.97)^b,e^	**0.007**
Cord blood (N=200)^$^					
	**SARS-CoV-2 antisera positive**		**SARS-CoV-2 antisera negative**		***p***
	**Anaemic** ^#^ **(n=70)**	**Normal (n=49)**	**Anaemic** ^#^ **(n=56)**	**Normal (n=25)**
RDW (%)	15.51 (2.15)^a,b.c^	14.83 (0.76)^a^	14.78 (0.84)^b^	14.63 (1.14)^c^	**0.008**
PDW (%)	18.28 (0.94)^a,b^	18.54 (1.02)	18.81 (1.19)^a^	18.93 (1.24)^b^	**0.015**
Newborn outcome (N=165)^$^					
	**Anaemic** ^#^ **(n=58)**	**Normal (n=39)**	**Anaemic** ^#^ **(n=44)**	**Normal (n=24)**	***p***
Newborn length (cm)	48.52 (2.79)^a^	48.85 (2.43)	50.16 (2.32)^a^	48.75 (2.09)	**0.009**

The mean values were compared between groups and within groups by One-way ANOVA. Only variables where F values were significant by ANOVA are shown here. Same alphabets in the superscript are given to denote significant difference within these groups upon Bonferroni’s post hoc multiple comparison. ^#^Groups based on maternal anemia status of the mother. ^$^12 of 212 cord blood samples had clots and analysis failed. Total missing data of a few parameters of the newborn outcome is n=47, of which fetal/neonatal death accounted for n=10. white blood cells count, RBC, red blood cells count; MCV, mean corpuscular volume; MCH, mean corpuscular haemoglobin; MCHC, mean corpuscular hemoglobin concentration; PCV, packed cell volume; RDW, red cell distribution width; PDW, platelet distribution width; Lymp.absol, absolute lymphocyte count; Neutro.absol, absolute neutrophil count; PLR, platelets to lymphocytes ratio; NLR, neutrophil to lymphocytes ratio. Values in bold signifies p<0.05.

### 3.4 Placental variables

**Figure 1:**
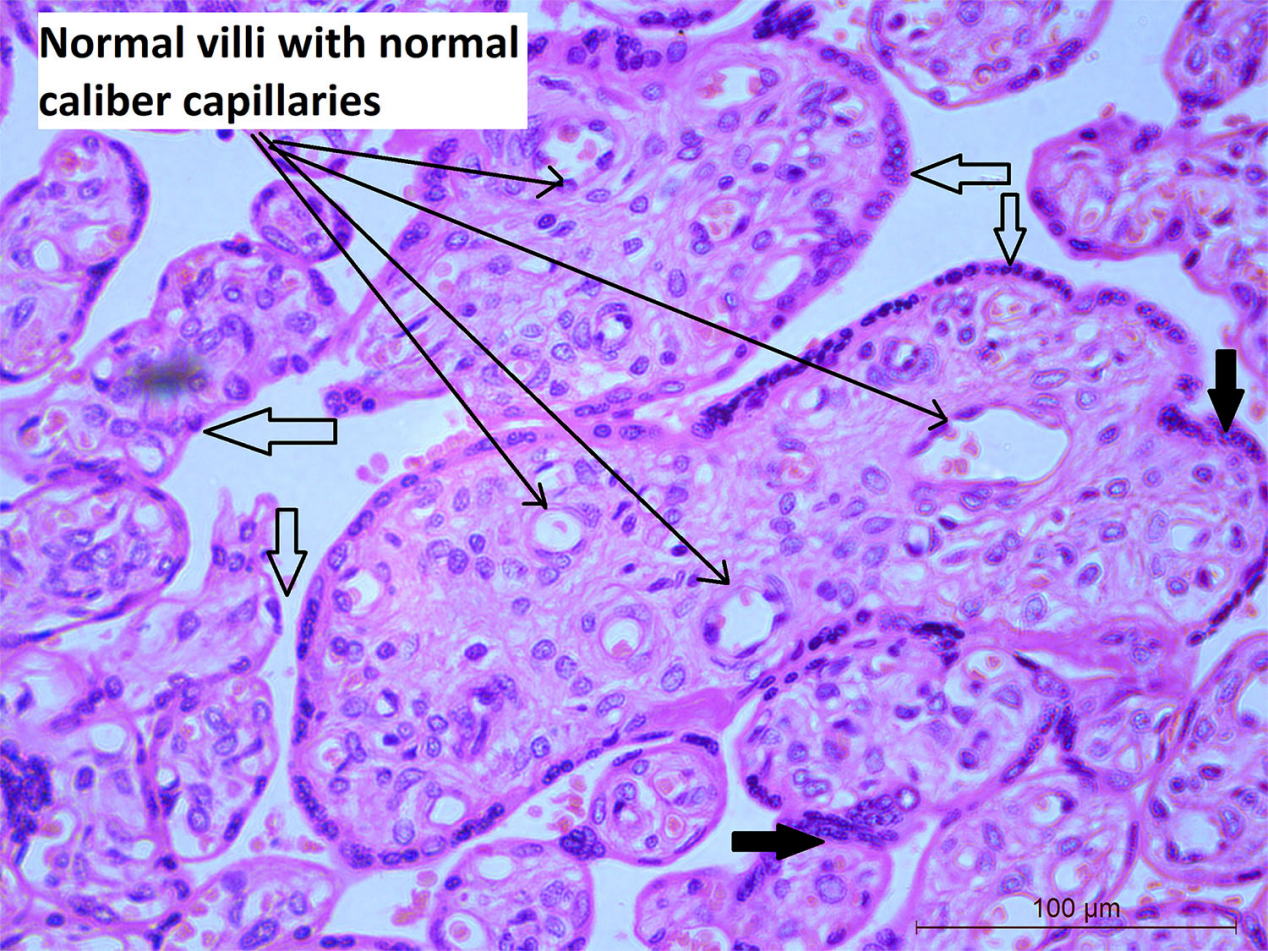
Histology of villi from normal placenta: Microphotograph shows normal chorionic villi (empty bold arrows) from placenta of mother who was COVIID-19 negative and non-anemic. The villi are lined by trophoblast cells (black bold arrows) with few scattered normal capillaries in the stroma of the villi (long black arrows). Original magnification, ×20.

**Figure 2:**
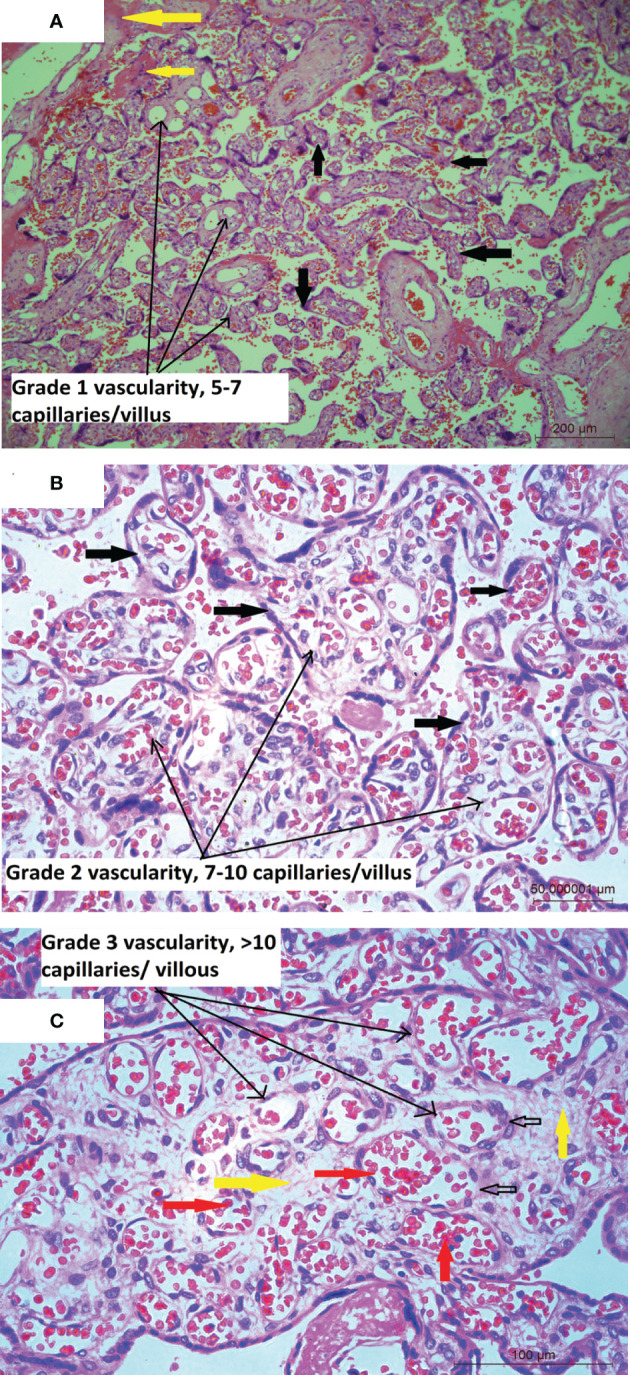
**(A)** Placental histology showing increased number of blood vessels: Microphotograph from placenta of COVID-19 positive mother shows chorionic villi (bold black arrows) with mild increase in the number of capillaries (5-7/villus) in the stroma (long black arrows), with normal amount of fibrin (bold yellow arrows). Original magnification, ×10. **(B)** Microphotograph shows chorionic villi from placenta of COVID-19 positive mother. The villi are marked in bold black arrows, are lined with trophoblast cells and show moderate or Grade 2 increase in the number of capillaries (7-10/villus) containing red blood cells (RBCs) in the stroma (long black arrows) of majority of the villi. Original magnification, ×20. **(C)** Microphotograph shows chorionic villus from placenta of COVID-19 positive mother showing Grade 3 increase in the number of capillaries per villus in the stroma (bold yellow arrows) of the villi (long black arrows), bold black empty arrows point to capillaries lined by endothelial cells and containing RBCs (bold red arrows). Original magnification, ×20.

**Figure 3:**
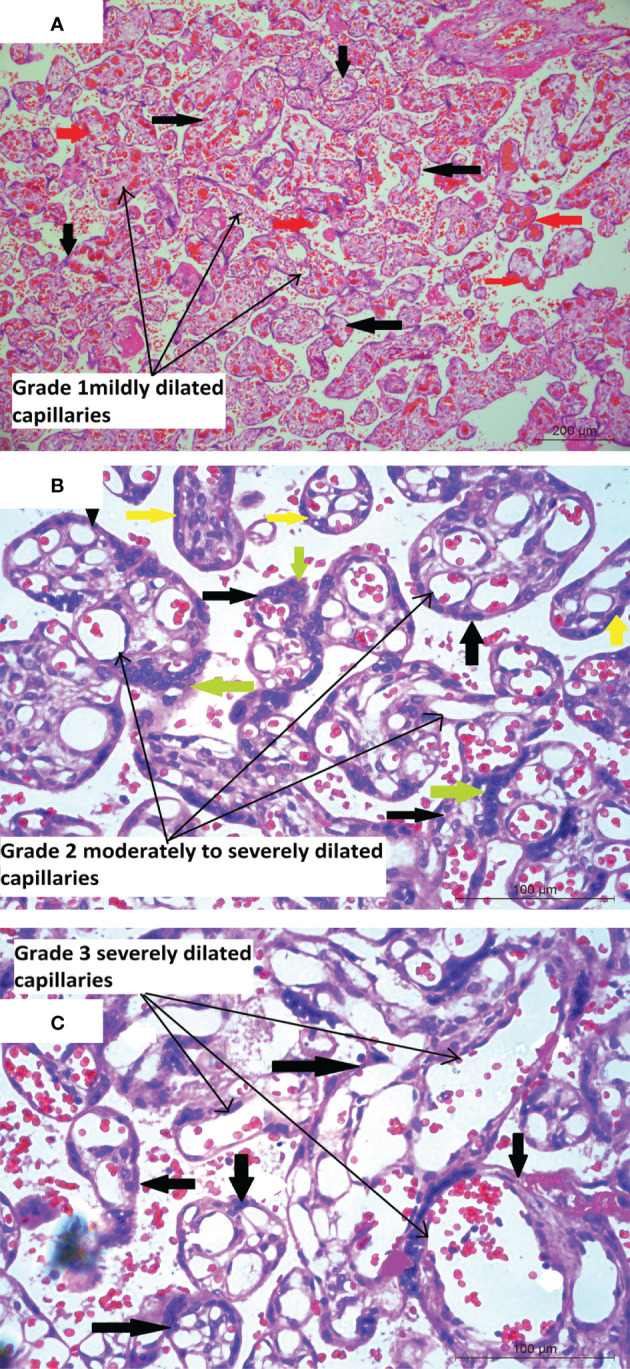
**(A)** Placental histology showing dilated blood vessels Microphotograph shows multiple chorionic terminal villi (bold black thick arrows) from placenta of COVID-19 positive mother with the stroma containing mildly dilated and congested capillaries (long black arrows). The congested capillaries with RBCs are marked in bold red arrows. Original magnification, ×10. **(B)** Microphotograph shows multiple chorionic terminal villi (bold black thick arrows) from placenta of COVID-19 positive mother with the stroma containing moderately to severely dilated capillaries (long black arrows) occupying most of the villus area. The villi are lined by syncytiotrophoblasts (bold green arrows) and cytotrophoblasts (bold yellow arrows). Original magnification, ×20. **(C)** Microphotograph shows chorionic terminal villi (bold black thick arrows) lined by syncytiotrophoblasts and cytotrophoblasts, from placenta of COVID-19 positive mother with extremely dilated fetal capillaries (long black arrows) occupying a major part to the whole of the villus area. Original magnification, ×20.

**Figure 4:**
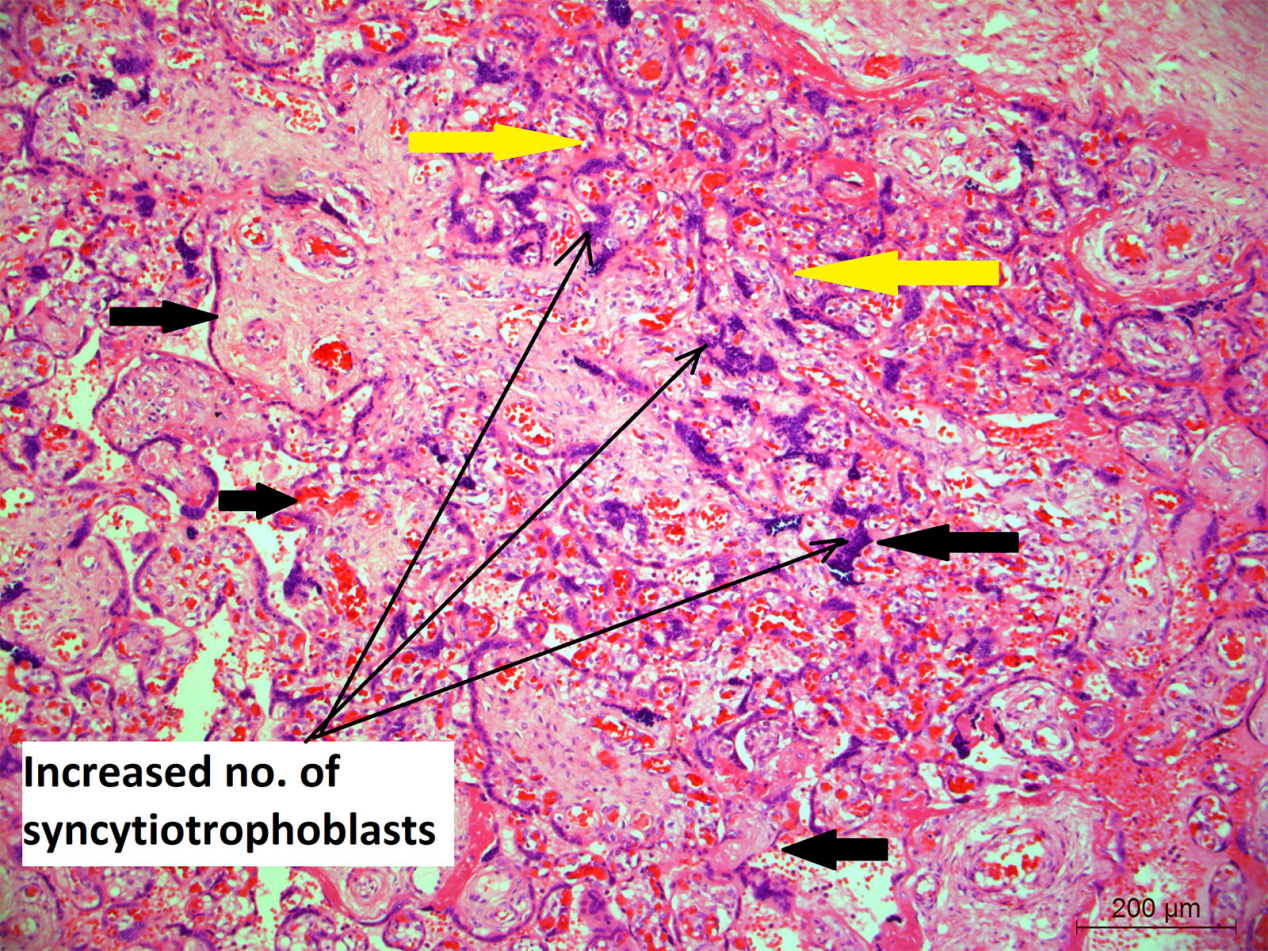
Placental histology showing increased number of syncytiotrophoblasts; Microphotograph shows chorionic villi (bold black thick arrows) from placenta of COVID-19 positive mother lined by increased number of multinucleated syncytiotrophoblasts (long black arrows). Moreover, the bold yellow arrows also show the area where the villi are closely placed indicating villus agglutination. Original magnification, ×10.

**Figure 5:**
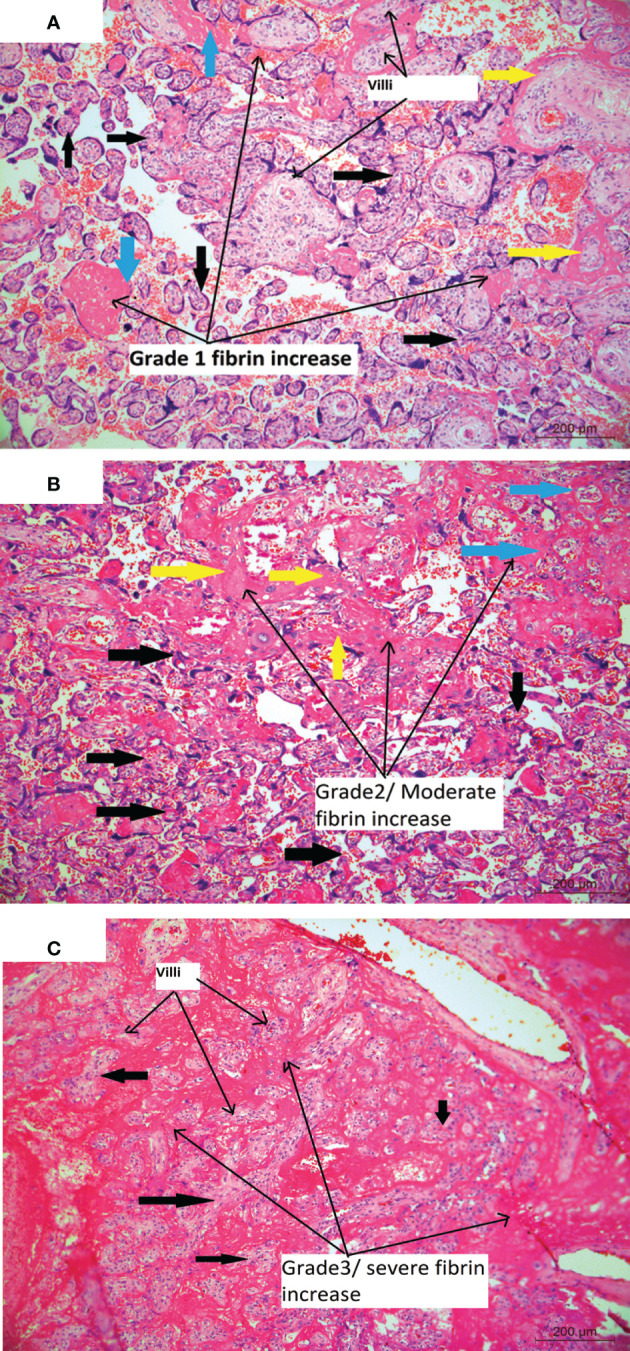
**(A)** Placental histology showing varied deposition of fibrin: Microphotograph shows chorionic villi (bold black thick arrows) from placenta of COVID-19 positive mother and with mild and grade1 increase in fibrin deposition in between (intervillous/blue bold arrows) and around the villi (perivillous/bold yellow arrows) (long black arrows) while the normal villi are shown in short black arrows. Original magnification, ×10. **(B)** Microphotograph showschorionic villi (bold black thick arrows) from placenta of COVID-19 positive mother and with moderate and grade2 increase in fibrin deposition in between (intervillous/bold yellow arrows) and around the villi (perivillous/bold blue arrows) and long black arrows. Original magnification, ×10. **(C)** Microphotograph shows multiple chorionic villi chorionic villi (bold black thick arrows) from placenta of COVID-19 positive mother, majority of which are surrounded by excessive amount of fibrin (intravillous and intervillous) (long black arrows) while the villi are shown in short black arrows. Original magnification, ×20.

Mean placental weight (**Table 1**) was relatively lower in seropositive group but the difference was not significant. Histopathological features were studied in all the 212 placentae received. **Figure 1** shows a typical histology of placentae from seronegative and non-anaemic healthy mothers, where the normal sized chorionic villi are seen lined by normal syncytiotrophoblasts and cytotrophoblasts and with the stroma showing normal fetal capillaries. When compared between the groups, acute mild chorioamnionitis was observed to be significantly higher in the membranes of seropositive placentae. Most of the umbilical cords (UC) were histologically normal in both seropositive and negative groups. With respect to placenta proper, histological features, like mild to severe degree of hypervascularity or increased vascularity of villi (increased number of fetal capillaries/villus) (**Figures 2A–C**), increased dilatation of capillaries in the villi (**Figures 3A–C**) and increased number of syncytiotrophoblasts with the formation of syncytial knots (**Figure 4**) and increased fibrin deposition (**Figures 5A–C**) were observed in seropositive placentae. Other features like villitis (**Figure S2**), vasculitis involving blood vessels in the chorionic villi, intervillous hemorrhage (**Figure S3**), infarcts in the decidua as well as the villi, smaller terminal villi indicating accelerated villous maturation (**Figures S4, S5**), calcification, decidual inflammation, villous agglutination and avascular fibrosed villi (**Figure S5**) were also found to be significantly more in the seropositive placentae.

**Table 4** shows the difference in various histopathological findings between the placentae of seropositive and seronegative mothers and the association was tested using Chi-square test. There were significant increase in hypervascularity of villi of grade 3 category (28 vs. 11%), dilated blood vessels in the villi of grade 2 (84.9 vs. 36.3%), number of syncytiotrophoblasts (84.8 vs. 69.2%), intervillous haemorrhage (40.3 vs. 15.4%), degeneration of trophoblast lining (39.5 vs. 5.5%) and villus agglutination (52.5 vs. 17.6%) in placental sections from seropositive group when compared to the negative group.

**Table 4:** Comparison of placental histopathological features based on SARS-CoV-2 serological status of the mother.

Histological variable	Grade	Seropositive, n (%)	Seronegative, n (%)	*p*
Umbilical cord	Normal	118 (98.3)	90 (98.9)	1.0
	AI^#^	2 (1.7)	1 (1.1)	
Membranes (chorion and amnion)	Normal,	44 (36.7)	55 (60.4)	**0.01**
	AC^$^ Grade1	59 (49.2)	30 (33)	
	AC Grade 2	12 (10)	5 (5.5)	
	AC Grade 3	5 (4.2)	1 (1.1)	
Hypervascularity of villi	Absent	3 (2.5)	17 (18.7)	**0.000**
	Grade1	10 (8.4)	28 (30.8)	
	Grade 2	72 (60.5)	36 (39.6)	
	Grade 3	34 (28.6)	10 (11)	
Dilated blood vessels in villi,	Normal	5 (4.2)	16 (17.6)	**0.000**
	Grade 1	13 (10.9)	42 (46.2)	
	Grade 2	101 (84.9)	33 (36.3)	
Number of syncytiotrophoblasts	Grade 1	18 (15.2)	28 (30.8)	**0.02**
	Grade 2	101 (84.8)	63 (69.2)	
Fibrin	Normal,	71 (59.7)	58 (63.7)	0.44
	Grade 1	18 (15.1)	18 (19.8)	
	Grade 2	21 (17.6)	11 (12.1)	
	Grade 3	9 (7.6)	4 (4.4)	
Villitis	Absent	106 (89.1)	88 (96.7)	0.06
	Present but focal	13 (10.9)	3 (3.3)	
Vasculitis	Absent	98 (82.4)	89 (97.8)	**0.000**
	Present	21 (17.6)	2 (2.2)	
Intervillous haemorrhage	Absent	71 (59.7)	77 (84.6)	**0.000**
	Present	48 (40.3)	14 (15.4)	
Infarcts	Absent	107 (89.9)	87 (95.6)	0.19
	Present	12 (10.1)	4 (4.4)	
Accelerated villous maturation	Absent	58 (48.7)	67 (73.6)	**0.000**
	Present	61 (51.3)	24 (26.4)	
Calcification	Absent	71 (59.7)	68 (74.7)	**0.03**
	Present	48 (40.3)	23 (25.3)	
Decidual inflammation	Absent	97 (82.2)	86 (94.5)	**0.01**
	Present	21 (17.8)	5 (5.5)	
Degeneration of trophoblasts lining villi	Absent	72 (60.5)	86 (94.5)	**0.000**
	Present	47 (39.5)	5 (5.5)	
Villus agglutination	Absent	57 (47.5)	75 (82.4)	**0.000**
	Present	63 (52.5)	16 (17.6)	
Fibrosed avascular villi	Absent	104 (86.7)	88 (96.7)	**0.01**
	Present	16 (13.3)	3 (3.3)	

Chi-square test was performed to study the association between different histological variables and serological status. Significant difference (p<0.05) are given in bold. ^#^AI, Acute inflammation- Infiltration with polymorphs. ^$^AC, Amniochorionitis.

We further analyzed between anemic mothers within seropositive and negative groups based on maternal anemia status (**Table 5**) to find out that chorangiosis (*p*=0.000), dilated blood vessels (*p*=0.000), syncytiotrophoblasts ((*p*<0.01), accelerated villous maturation were significantly higher (*p*=0.02) in seropositive anemic mothers. We tested but found no difference in fibrin, villitis, karyorrhexis, infarcts, fibrosed, avascular villi, premature villi and other parameters, while weaker association was found for calcification and decidual inflammation,

**Table 5:** Comparison of placental histopathological features based on SARS-CoV-2 serological status of the mother and maternal anemia.

Histological variable	Severity	SARS-CoV-2 positive		SARS-CoV-2 negative		*p*
		Anaemic n (%)	Normal n (%)	Anaemic n (%)	Normal n (%)	
Chorangiosis	Absent/mild (n=56)	9 (4.3)	4 (1.9)	26 (12.5)	17 (8.1)	**0.000**
	Moderate/severe (n=152)	61(29.3)	45 (21.6)	36 (17.3)	10 (4.8)
DB vessels	Absent/mild (n=74)	10 (4.8)	6 (2.8)	37(17.8)	19 (9.1)	**0.000**
	Moderate/severe (n=134)	58 (27.9)	43 (20.7)	25 (12.0)	8 (3.8)
Syncytiotrophoblasts	Absent/mild (n=74)	21(10.1)	11 (5.2)	26 (12.5)	16 (7.7)	**0.006**
	Moderate/severe (134)	49 (23.5)	38 (18.3)	36 (17.3)	11 (5.2)
Villous degeneration	Absent (n=157)	43 (20.7)	29 (13.9)	58 (27.9)	27 (13)	**0.000**
	Present (n=51)	27 (13)	20 (9.6)	4 (1.9)	0
IBV	Absent (n=185)	58 (27.9)	40 (19.2)	61 (29.3)	26 (12)	**0.006**
	Present (n=23)	12 (5.8)	9 (4.3)	1 (0.5)	1 (0.5)
Intervillous hemorrhage	Absent (n=146)	41 (19.7)	30 (14.4)	51 (24.5)	24 (11.5)	**0.002**
	Present (n=62)	29 (13.9)	19 (4.3)	11 (5.2)	3 (1.4)
Villus agglutination	Absent (n=132)	35 (16.7)	22 (10.6)	50 (23.9)	25 (11.9)	**0.000**
	Present (n=76)	36 (17.2)	27 (12.9)	12 (5.7)	1 (0.5)
Small terminal villi	Absent (n=124)	28 (13.5)	30 (14.4)	44 (21.1)	22 (10.6)	**0.000**
	Present (n=84)	42 (20.2)	19 (9.1)	18 (8.6)	5 (2.4)

Chi-square test was performed to study the association between different histological abnormalities with SARS-CoV-2 serology based on maternal anemia status. Dilated blood (DB) vessels, IBV inflammation around blood vessels. Values in bold signifies p<0.05.

### 3.5 Multivariate logistic regression analysis of risk factors for abnormal placental findings

Among placental histopathological findings, logistic regression models (**Table 6**) were developed for moderate to severe chorangiosis, dilated blood vessels, syncytiotrophoblasts, agglutination of villus and calcification (data not shown) as dependent variables. The predictors or independent variables were included in the models were based on either their significant association in bivariate analyses or based on assumptions of their potential to influence the outcome. In all the models, SARS-CoV-2 seropositivity was an independent risk factor for increased chorangiosis (AOR=8.74; 95% CI 3.51 - 21.76, *p*<0.000), dilated blood vessels (AOR=12.7;95% CI 5.46 - 29.75,*p*<0.000), syncytiotrophoblasts (AOR=2.86; 1.36 - 5.99, *p*<0.005), villus agglutination (AOR=9.27, 95% CI 3.68-23.32, *p*<0.000) and calcification (AOR=2.72; 95% CI 1.21-6.11, *p*<0.015) after adjustments for the other covariates. In separate models (data not shown) sociodemographic and maternal factors (maternal age, BMI, income, parity, gestational age), haematological factors (haemoglobin, WBC, PCV, MCV, RDW, Neutrophils, Lymphocytes, Monocytes, NLR, PLR) and birth weight were individually tested along with SARS-CoV-2 seropositivity, but none of the parameters contributed significantly. In addition, we will like to emphasize that haemoglobin did not emerge as a risk factor for any of these histopathological outcomes tested. Advancing gestational age showed a trend of increasing risk (AOR=8.97, *p*=0.057) for severe syncytiotrophoblasts, but was not significant for the other placental abnormalities. Low birth weight was found to be associated with villus agglutination (AOR=3.72,*p*=0.012). Abnormal WBC count (other than 4-12×10^3^/µl) was associated with higher risk of severe chorangiosis (AOR=2.76, *p*=0.031), syncytiotrophoblasts (AOR=2.96, *p*=0.041) and increased agglutination of villus (AOR=2.33, *p*=0.028).

**Table 6:** Bivariate and multivariate logistic regression models for SARS-CoV-2 serological status as a risk factor for placental abnormality.

Dependent variable	Independent variable				Model 1^a^			Model 2		
Placental abnormality	SARS-CoV-2 serology	COR	95% CI	*p*	AOR	95% CI	*p*	AOR	95% CI	*p*
Chorangiosis None/mild vs. moderate/severe	Negative	1			1			1		
	Positive	8.20	3.66-18.37	0.000	8.74	3.51- 21.76	0.000	8.04 ^b^	3.55-18.24	0.000
Dilated blood vessels None/mild vs. moderate/severe	Negative	1			1					
	Positive	12.42	5.75-26.82	0.000	12.74	5.46 - 29.75	0.000			
Syncytiotropho blasts None/mild vs. moderate/severe	Negative	1			1			1		
	Positive	2.53	1.30-4.89	0.006	2.86	1.36-5.99	0.005	2.62^c^	1.34-5.14	0.005
Villus agglutination None/mild vs. moderate/severe	Negative	1			1			1		
	Positive	5.32	2.57-11.02	0.000	9.27	3.68 -23.32	0.000	5.46^d^	2.54-11.73	0.000

a Variables entered on step 1: Age, BMI, Income, Parity, GA, WBC, PCV, MCV, RDW, Neutrophils, Lymphocytes, Monocytes, NLR, PLR, birth weight, Haemoglobin, SARS-CoV-2 group.

b Variables entered on step 1: SARS-CoV-2 group and WBC.

c Variables entered on step 1: SARS-CoV-2 group and GA.

d Variables entered on step 1: SARS-CoV-2 group, GA, birth weight and WBC.

COR, crude odds ratio; AOR, adjusted odds ratio; CI, confidence interval; GA, gestational age; NLR, Neutrophil to Lymphocyte ratio; PLR, Platelet to Lymphocyte ratio, GA, gestational age. Values in bold signifies p<0.05.

## 4 Discussion

In the first global wave of COVID-19, India saw its first outbreak between March, 2020 and February, 2021, later followed by the emergence of the Delta variant (B.1.617.2), responsible for India’s deadly second wave following its emergence in February 2021 (16, 30). In the present study, we observed that, whilst all of the participants were RT-PCR negative for SARS-CoV-2 virus at the time of hospital admission for delivery during August-November, 2021, and whilst almost all of them self-reported to have never experienced COVID-19 like symptoms during pregnancy, but about 58% were carrying IgG antibodies against SARS-CoV-2. The national sero-surveillance data of India showed SARS-CoV2 IgG prevalence as 24.1% [95% CI (23.0 - 25.3)] in the general population during December, 2020- January, 2021, before the vaccines were available (31). By June-July, 2021, this value had increased to 62.3% [95% CI (60.9 - 63.7)] in unvaccinated adults (including pregnant women). Vaccine roll out started for pregnant women in our country almost at the end of October 2021 (and our study cohort included pregnant women till November 2021) due to which we say that all the women were unvaccinated. Sero-prevalence was significantly higher among individuals who had received either one or two vaccine doses (81.0-89.8%) (32). A meta-analysis of all sero-prevalence data in India between March 2020 to August 2021 showed an overall pooled SARS-CoV-2 IgG sero-prevalence of 20.7% [95% CI (16.1 - 25.3)] in the first and 69.2% [95% CI (64.5 - 73.8)] in the second wave (33). Therefore, it is highly likely that majority of our subjects were infected by SARS-CoV2 during their gestational period, lying between November, 2020 and November, 2021, irrespective of the SARS-CoV2 variant involved. A number of serology studies globally reported a reduced immunological response (34) and rapid decay of the antibody (35) in asymptomatic cases or in mild COVID-19 infection (36).Others also reported a longer duration of IgG in circulation lasting beyond six months (37) to twelve months as in convalescent plasma donors having higher titer of antibodies (38). Since our study was conducted before COVID-19 vaccination drive for pregnant women in India, the presence of SARS-CoV-2 IgG only indicates asymptomatic infection in the past, most likely due to an infection in the preceding 9-months of gestational period. In another study, the authors reported that about 84% of their infected pregnant subjects were asymptomatic and such that the duration of the placental histopathological findings could not be determined (39).

The prevalence of COVID-19 infection in our study was much higher when compared to the studies by Bachani et al. (40) of 16.3%, and of Waghmare et al. (41) showing 12.3%, or of Singh et al. (42) reporting 4.83%. Since all of these studies estimated prevalence based on SARS-CoV-2 antigen and therefore active infection, and likely symptomatic patients only, the values are not comparable to our values of seropositivity. Our finding corroborates with other reports stating the fact that COVID-19 causes milder disease in pregnant women (40, 43–45). In our study population, however, birth weight and APGAR scores (appearance, pulse, grimace, activity, and respiration) of all neonates were within normal limits when compared between the seropositive and seronegative mothers, and no obvious adverse effect of COVID-19 was reported on these neonatal outcomes as reported earlier (40, 44, 45). A few other studies had reported lower birth weight in SARS-CoV-2 infected pregnant women (43, 46, 47) but it was found similar in the two groups in our participants.

Anemia has been stated to be one of the co-morbidities affecting COVID-19 pregnancies and in India anemia in pregnancy is very common. Earlier studies have reported development of anemia in COVID-19 patients and the patients presented with decreased Hb and increased ferritin levels (21, 48). However, in our study population, hemoglobin level was similar in seropositive and negative groups among non-anemic women as reported in a few earlier studies (48, 49). Also, ferritin level in both maternal and cord blood were similar between the seropositive and negative groups, likely because none of our subjects were suffering from an acute infection during delivery. It was however, interesting to note a lower ferritin level in the anemic seronegative subjects, but showing higher trend in anemic seropositive subjects, which maybe due to a non-acute COVID-19 infection. This contrasts with other studies where asymptomatic women with RT-PCR positive acute infection had a significantly higher level of ferritin in COVID-19 positive group (266.4 μg/l and 40.5 μg/l, *p*=0.001) along with higher C-reactive protein compared to COVID-19 negative group (50).We observed higher leucocytosis, lymphocytosis, and neutrophilia in seropositive mothers similar to other studies (1, 51), but not all (48). Neutrophil lymphocyte ratio (NLR) and platelet lymphocyte ratios (PLR) were lower in seropositive mothers in our study, which could be due to the presence of both leucocytosis and lymphocytosis leading to their lower levels, while others have reported lymphopenia as the leading cause behind elevated NLR and PLR. Dixon JB et al. (52) have showed that WBC, neutrophils and lymphocyte counts increase with increasing BMI and decrease with age, while another study (53) reported age, race, and obesity to be significantly associated with the WBC count in healthy individuals. In the present study, the mean age of the participating pregnant mothers was less than 25 years and mean BMI was higher (>24 kg/m^2^), which could be the reason for high WBC counts in our participants. NLR is known to be a steady marker for systemic inflammation, which is less affected by physiological conditions, increase in patients suffering from severe COVID-19 infection, and linked to a poor outcome (54). However, the data comparing NLR in pregnant mothers who are healthy with SARS-CoV-2 infected women are limited and results are inconsistent. In our population, we failed to notice striking difference in NLR among the groups, although in regression analysis, normal NLR seemed to offer protection from adverse histopathological events.

To date, in placental histopathology in COVID-19 (25–27, 55, 56) specific histologic features or hallmarks of the disease have not been identified. Placental histopathology in our study revealed a few intriguing features which were difficult for us to decipher due to the paucity of literature. The predominant finding was extreme dilatation of villous vessels or the villous capillaries, which is one of the features of fetal vascular malperfusion (FVM), leading to extreme thinning of the villous matrix, distortion of the villous outline, and disruption of outer trophoblast layer, seen in both terminal and stem villi, present in mostly in COVID-19 seropositive cases, irrespective of maternal anaemia status. Capillary dilatation was accompanied by villous hypervascularity (increased number of capillaries per villus), in proportion to vessel dilation. Pathological evidence of FVM was earlier shown in many different studies (12, 25, 27, 44, 57–62), however, the severity of changes observed in our study was not seen in other studies.

Since both anemia and COVID-19 are known to be causes of maternal hypoxia, we wanted to examine the effect of maternal anemia on COVID-19-affected placentas or vice versa. In our study, a significant increase in syncytiotrophoblasts (STBs) and syncytial knots in villi was observed in placentas of anemic COVID-19 seropositive mothers, similar to earlier studies (59, 60). Syncytial knots usually develop in hypoxic placenta leading to increased terminal villi vessels as a compensatory response. Hypoxia due to maternal anemia has been reported to significantly increase terminal villi blood vessels (59, 60) and SARS-CoV-2 infection is also known to cause maternal hypoxia, probably due to a hypercoagulable state, leading to hypoperfusion in the placenta and subsequent hypoxic-ischemic injury to the placenta (63). However, the exaggerated villous capillary response observed in our study could probably be explained by placental hypoxia attributed to COVID-19 solely with limited contribution by anemia.Apart from hyper mature villi which were significantly observed in COVID seropositive anemic cases, all the remaining parameters, including dilated fetal vessels, although slightly increased number in COVID seropositive anemic cases, but were not significantly different from the non-anaemic group, thus pointing to a lesser effect of anemia on fetal vascularity in comparison to COVID-19.

We had also observed maternal vascular malformations (MVM), like perivillous fibrin, infarcts, agglutination of villi, accelerated villous maturation (compensatory change due to MVM, composed of short hyper mature villi for gestational age), intervillous hemorrhage significantly higher in seropositive mothers. With respect to FVMs and MVMs, there are conflicting reports in the literature. Prabhu (64) had reported a higher incidence of lesions about FVM in the COVID cases while other studies (27, 65) reported a higher frequency of MVMs than controls. However, both the studies dealt with placentas from preterm pregnancies in which MVM lesions are commonly known. In contrast, we had studied placentas from term deliveries.

The histological features of hypoxia we observed were moderate to severe increase (chorangiosis) as well as dilation of capillaries, indicating feto-vascular malformation in placental villi and these were present in seropositive placentas irrespective of anemia. What was of greater clinical concern in our study was the fact that women were asymptomatic while the hypoxia induced changes in the placenta suggests “silent hypoxia” and possibilities of fetus being exposed to chronic fetal hypoxia (24, 66–68) and adverse fetal outcomes like pre-term birth, stillbirth, hypoxia of the fetal brain. We did not notice any profound impact of COVID-19 in terms adverse neonatal outcomes, such as birth weight, gestational age, admissions to ICU and APGAR scores as reported in the earlier studies, maybe pertaining to milder infection. However, a long-term effect of fetal exposure to chronic subclinical hypoxia due to abnormalities in the placentas cannot be ruled out and will need longitudinal follow up studies of the affected children.

## 5 Conclusion

Although the prevalence of anemia was high in the present study, its effects on placentae were less prominent than that of SARS-CoV-2. The most intriguing and novel finding of the study was strong evidences that of maternal COVID-19 infection, which was otherwise asymptomatic, was being associated with increased placental damage, indicating histopathological features of placental hypoxia and thus possibilities of intrauterine fetal hypoxia. The long-term adverse consequences of this placental pathology to the fetal and neonatal growth and development can be understood only through follow-up studies.

## 6 Strengths and limitations

This is one of the largest studies on pregnant women from this region examining effects of COVID-19 during pregnancy and placental histopathology. The cohort was exposed to one of the deadliest waves of COVID-19 in India and in spite of asymptomatic infection they developed severe placental histopathology. However, there are a few limitations of the study. Firstly, our observation was made in a single center and therefore, the findings cannot be generalized for the whole of the Indian population. Secondly, the cross-sectional study design did not allow us to assess the exact time of the time of SARS-CoV-2 infection, while the use of a self-reporting by recall method also failed as cases were asymptomatic. Further, we did not assess IgM sero-prevalence and hence the time of infection could not be established. Finally, maternal hemoglobin was measured only once at the time of hospital admission and hence it was not possible to determine if COVID-19 worsened the anemia status during pregnancy.

## Data availability statement

The raw data supporting the conclusions of this article will be made available by the authors, without undue reservation.

## Ethics statement

The studies involving human participants were reviewed and approved by Institutional Ethical Committee of National Institute of Nutrition (ICMR), registered with National Ethics Committee Registry for Biomedical and Health Research (NECRBHR), Department of Health Research, India. The patients/participants provided their written informed consent to participate in this study.

## Author contributions

MS was involved in conceptualization, methodology, validation, conducting research, writing manuscript, fund acquisition, and overall supervision. NS was involved in conducting research, recruitment of subjects and sample collection. NB performed the statistical analysis, PK was involved in the conception of the manuscript. KS performed hematological and histopathological work. JG helped in conducting the research. PS was involved biochemical analysis. BT in immunological analysis. RM was involved in conceptualization and data analysis. GR contributed to data analysis and edited the manuscript. GM was involved in sample procurement, validation of methodologies, data analysis, manuscript writing and editing. All authors contributed to the article and approved thesubmitted version.
