# Supplementary material for: Impact of COVID-19 during pregnancy on placental pathology, maternal and neonatal outcome – A cross-sectional study on anemic term pregnant women from a tertiary care hospital in southern India
Source: Front Endocrinol (Lausanne). 2023 Mar 21;14:1092104. doi: 10.3389/fendo.2023.1092104 (PMC10070875; doi:10.3389/fendo.2023.1092104)
Supplement: Supplementary file 1 [file DataSheet_1.docx]

**Supporting information**

1. **Annexure-I**

**QUESTIONNAIRE**

**Study No. 20-PT-02**

1. **ID No. of the patient** : _________________________________
2. **Name of the patient** : _________________________________
3. **Age** : ⬜ ⬜ yrs
4. **Height** : ⬜ ft ⬜ in
5. **Weight :** ⬜ ⬜ kgs
6. **Education status :** (1) Illiterate / (2) Schooling / (3) College ⬜
7. **Occupation :** (1) Working / (2) Not working ⬜
8. **Monthly income of family** : (1) < Rs. 5000 / (2) Rs. 5000-10,000 /

(3) Rs. 10,000-50,000 / (4) > Rs 50,000 ⬜

9) **Community**: (1) SC / (2) ST / (3) OBC / (4) OC / (5) Others ⬜

1. **Obstetric history :**

Gravida ⬜ Parity ⬜ No. of live children ⬜ ⬜

Age of children in years: (1) ⬜ ⬜ (2) ⬜ ⬜ (3) ⬜ ⬜

Previous history of still births: (1) Yes / (2) No ⬜

No. of children not surviving ⬜ and cause of death ____________________

History of spontaneous abortions: (1) Yes / (2) No ⬜

History of induced abortions: (1) Yes / (2) No ⬜

History of Pregnancy induced hypertension: (1) Yes / (2) No ⬜

History of Pregnancy induced Diabetes: (1) Yes / (2) No ⬜

History of earlier bleeding disorders: (1) Yes / (2) No ⬜

Place of previous deliveries: (1) Hospital or Nursing home / (2) Home ⬜

Nature of complications during pregnancies for recent births: ____________________

1. **Antenatal visits**: No. of visits: ⬜ (1) First trimester / (2) Second trimester / (3) Third trimester ⬜
2. **Immunisation history**:

Tetanus toxoid or TT given: (1) Yes / (2) No ⬜

If given then time of administration: (1) First trimester (2) Second trimester

(3) Third trimester ⬜

No. of doses : (1) Single (2) Two (3)Three ⬜

Any other vaccine given: (1) Yes / (2) No ⬜

If yes then name of the vaccine: ________

1. **History of intake of iron and folic acid supplements**: (1) Yes / (2) No ⬜

If Yes then tablets taken and dose **: ___________________________________**

1. **History of COVID-19 positivity** : (1) Yes / (2) No ⬜

If yes, RAT test positive ⬜ / RT-PCR positive ⬜

If yes, in (1) First trimester (2) Second trimester (3) Third trimester ⬜

If yes, (1) With symptoms / (2) No symptoms ⬜

If yes, Home isolation (1) Hospital admission (2) ⬜

If yes, Admitted to ICU: (1) Yes / (2) No ⬜

If yes, Treated with (1) Immunosuppressants (2) Antivirals (3) Symptomatic t/t ⬜

No. of COVID-19 vaccination doses taken : (1) Single (2) Two (3)Not taken ⬜

If yes, then taken in : (1) 1^st^ trimester (2) 2^nd^ trimester (3) 3^rd^ trimester (4) after delivery

1. **Clinical Symptoms at admission for delivery :**

Weakness / fatigue / malaise: 1 Yes / 2 No ⬜ ; Dyspnoea on exertion: 1 Yes / 2 No ⬜

Fever: 1 Yes / 2 No ⬜ ; Cough/cold: 1 Yes / 2 No ⬜

1. **Clinical signs at admission :**

Pulse: ________/ min ; Blood pressure: ________ mm/Hg

Respiratory rate:______________; Oxygen saturation : _________________

Palor in eyes and skin: (1) Yes / (2) No ⬜

Koilonychia: (1) Yes / 2 No ⬜ ; Jaundice: (1) Yes / (2) No ⬜

Pedal oedema: (1) Yes / (2) No ⬜

1. **COVID-19 positivity at admission**: (1) Positive (2) Negative ⬜

If yes, RAT test positive ⬜ / RT-PCR positive ⬜

1. **Newborn parameters :**

Weight: _____ kgs Crown-rump length: _______cms; Skin fold thickness: ______ cms

Head circumference: ________cms Mid-arm circumference: ________cms

1. **Final Diagnosis**: (Oligohydramnios/ pre-mature rupture of membranes/ pre-term birth/ IUGR/ still birth etc)-
2. **Annexure II**

**Anti- SARS-CoV-2 Antibody quantification in maternal and umbilical cord blood estimated by Enzyme-linked Immunosorbent Assay (ELISA)**

Antibodies against SARS-CoV-2 antigen were quantified using ELISA. ELISA plates were coated with Anti-SARS Cov-2 IgG Antibody and marketed as Covid Kavach^TM^ ELISA kit developed by ICMR-NIV, Pune, India and Zydus Diagnostics. 100 µl of positive and negative controls were added to the respective wells followed by 100 µl of diluted sample to each of the remaining wells and incubated for 1 hour at 37 ^0^C. After incubation 100 µl of anti-human IgG HRP enzyme reagent was added to each well, and incubated for 1 hour at 37 ^0^C which was followed by washing with 300 µl of 1x wash buffer (Model No: ALTA ELISA washer ADX100). Then 100 µl of substrate reagent was added to each well and the plate was kept in dark for 10 minutes at RT (Room temperature). Next, the reaction was stopped by adding 100 µl of stop solution and the signal was read at 450 nm in ELISA reader (Model No: BioTek Synergy HT).

Interpretation of the ELISA results: The performance of kit was calculated based on P/N ratio which was calculated by using the formula: P/N ratio=Average OD value of positive control/ Average OD value of negative control. The test was considered valid if the P/N ratio of positive control was greater than 1.5 and the cut off calculations was equal to average of negative control+0.2. For the test sample if OD value was > Cut-off value, the sample was considered as “Positive” and if OD value ≤ Cut-off value, sample was considered as “Negative”.

**Table S1: Spearman correlation between SARS-CoV-2 seropositive and seronegative mothers’ blood and cord blood**

| **Parameter** | **Between SARS-CoV-2 Seropositive mother’s blood & cord blood** | | **Between SARS-CoV-2 Seronegative Mother’s blood & cord blood** | |
| --- | --- | --- | --- | --- |
|  | **Correlation coefficient**  **(R-value)** | ***p*-value** | **Correlation coefficient**  **(R-value)** | ***p*-value** |
| **Hb (g/dl)** | 0.15 | 0.12 | 0.07 | 0.53 |
| **WBC (10^3^/µl)** | 0.06 | 0.51 | **0.35** | **0.001** |
| **MCV(fl)** | 0.17 | 0.06 | 0.11 | 0.31 |
| **Absolute lymphocyte count** | 0.07 | 0.46 | **0.23** | **0.03** |
| **Lymphocyte %** | -0.05 | 0.60 | 0.15 | 0.16 |
| **Absolute monocyte count** | 0.10 | 0.29 | 0.09 | 0.38 |
| **Monocyte %** | **0.37** | **0.000** | 0.13 | 0.27 |
| **Absolute neutrophil count** | -0.04 | 0.69 | **0.31** | **0.006** |
| **Neutrophil %** | -0.02 | 0.82 | **0.23** | **0.04** |
| **NLR** | -0.06 | 0.50 | **0.28** | **0.01** |
| **PLR** | -0.18 | 0.05 | 0.05 | 0.66 |
| **IgG**  **Antibodies** | **0.87** | **0.000** | **0.84** | **0.000** |

Footnote: Abbreviations: Hb- hemoglobin, WBC- White blood cells, MCV- Mean corpuscular volume, Neut. absolute-, Neut.- Neutrophils, NLR- neutrophils to lymphocyte ratio, PLR- platelets to lymphocytes ratio, IgG- Immunoglobulin. Numbers in bold indicate significant values at *p*<0.05.

**S1 Fig**


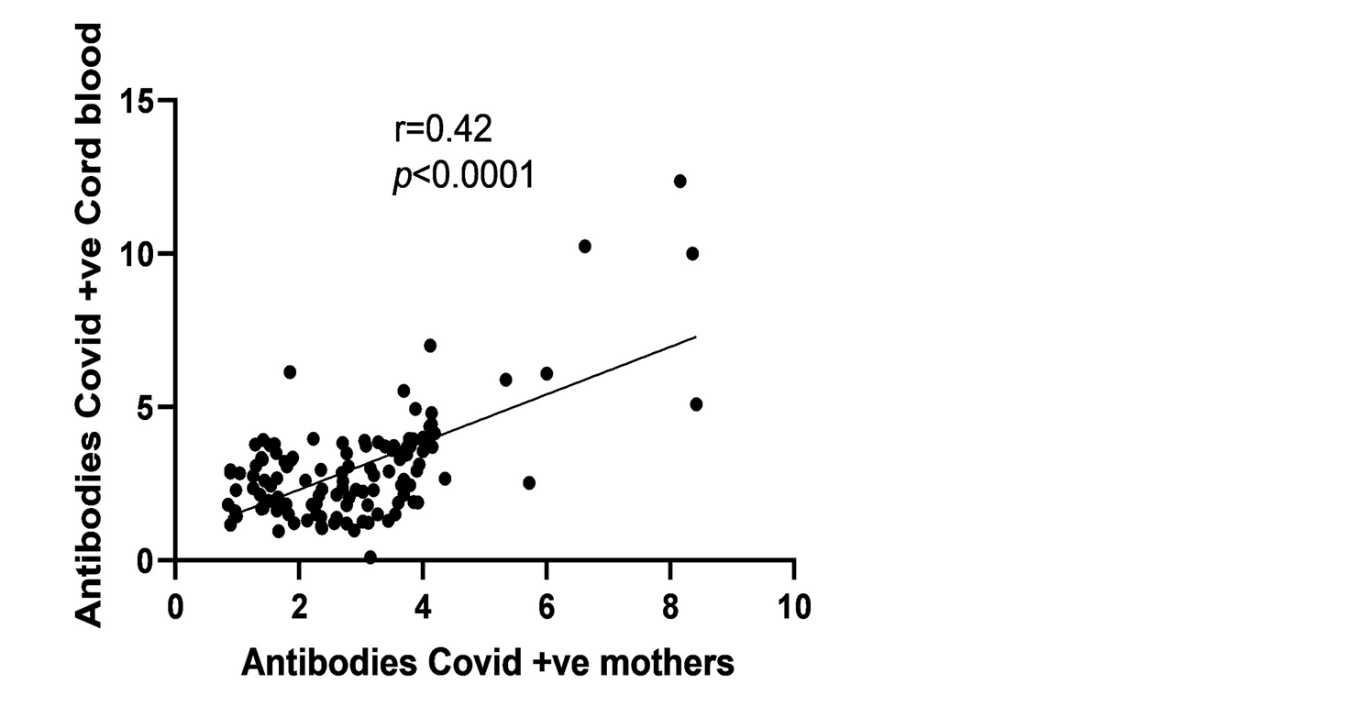


**S2 Fig**


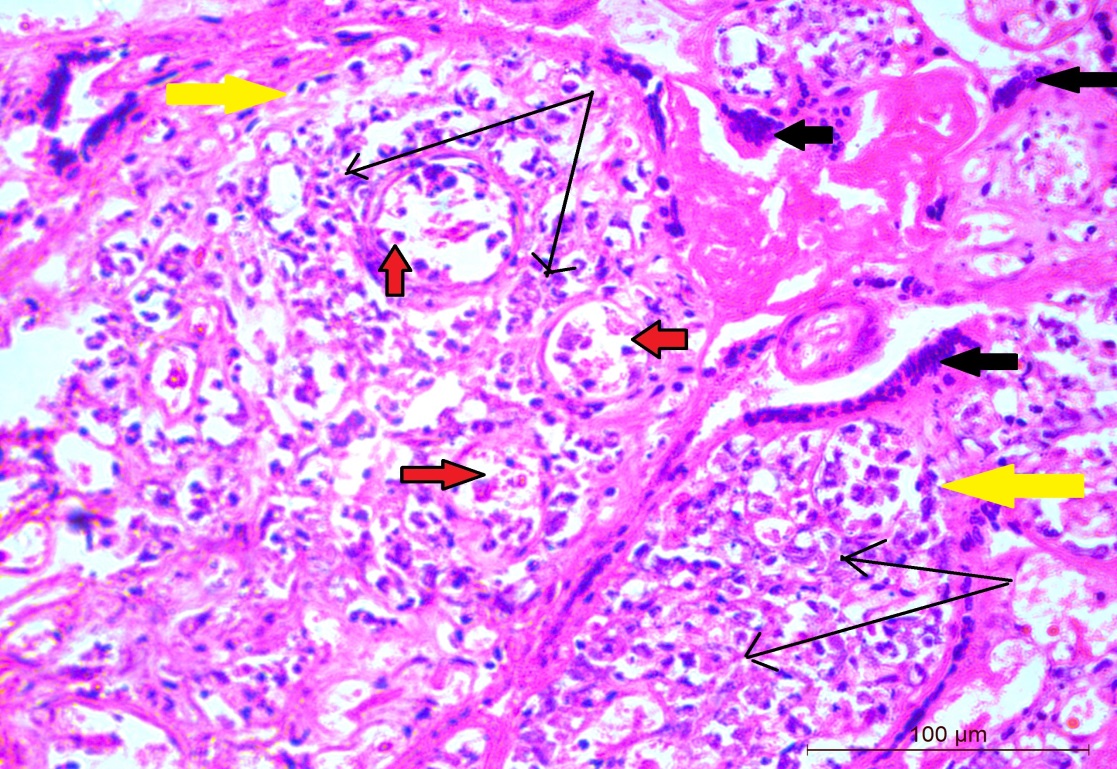


**S3 Fig**


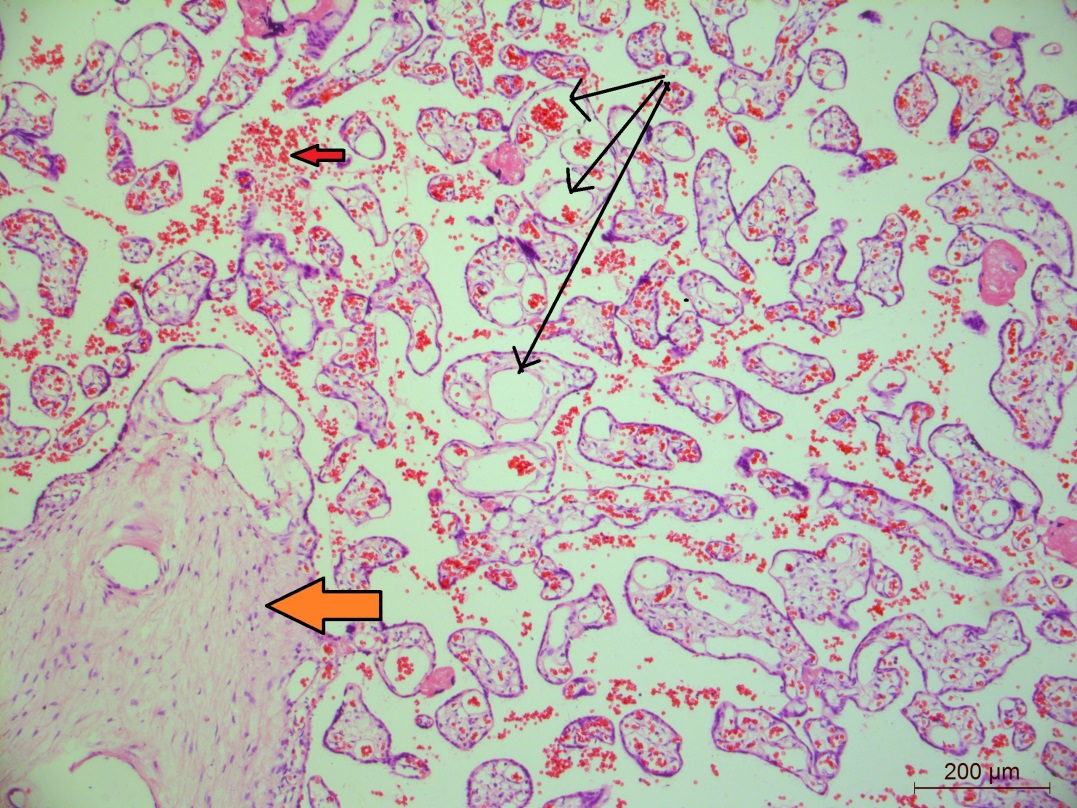


**S4 Fig**


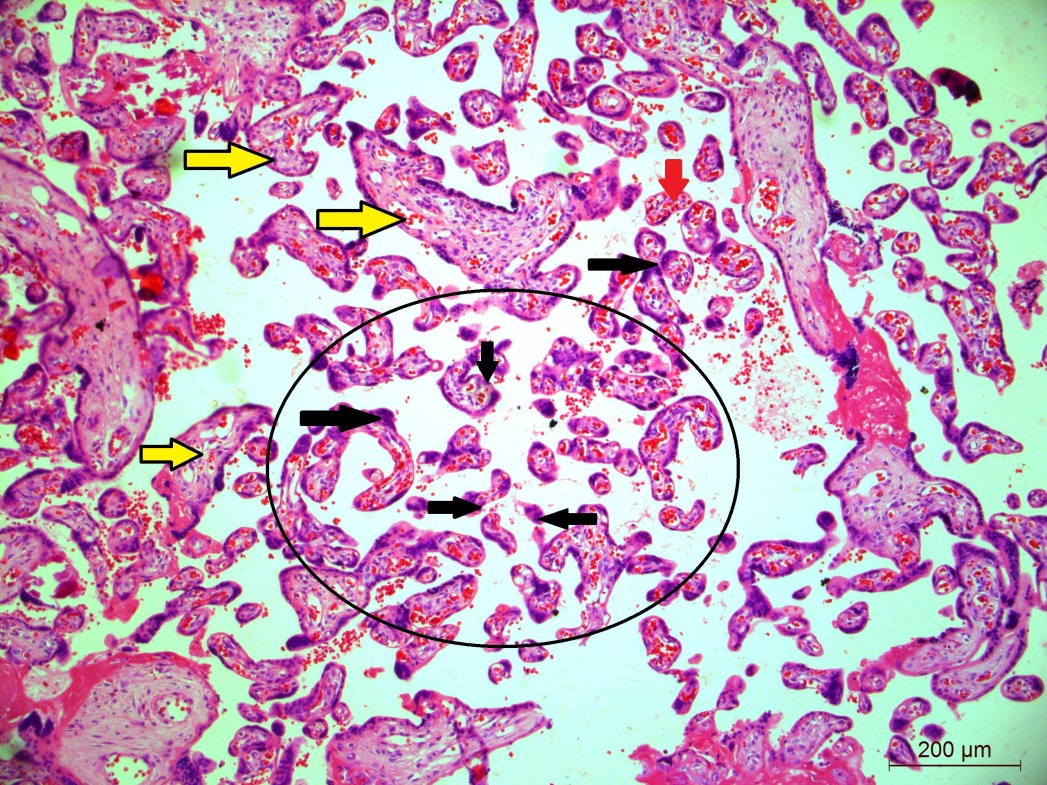


**S5 Fig**


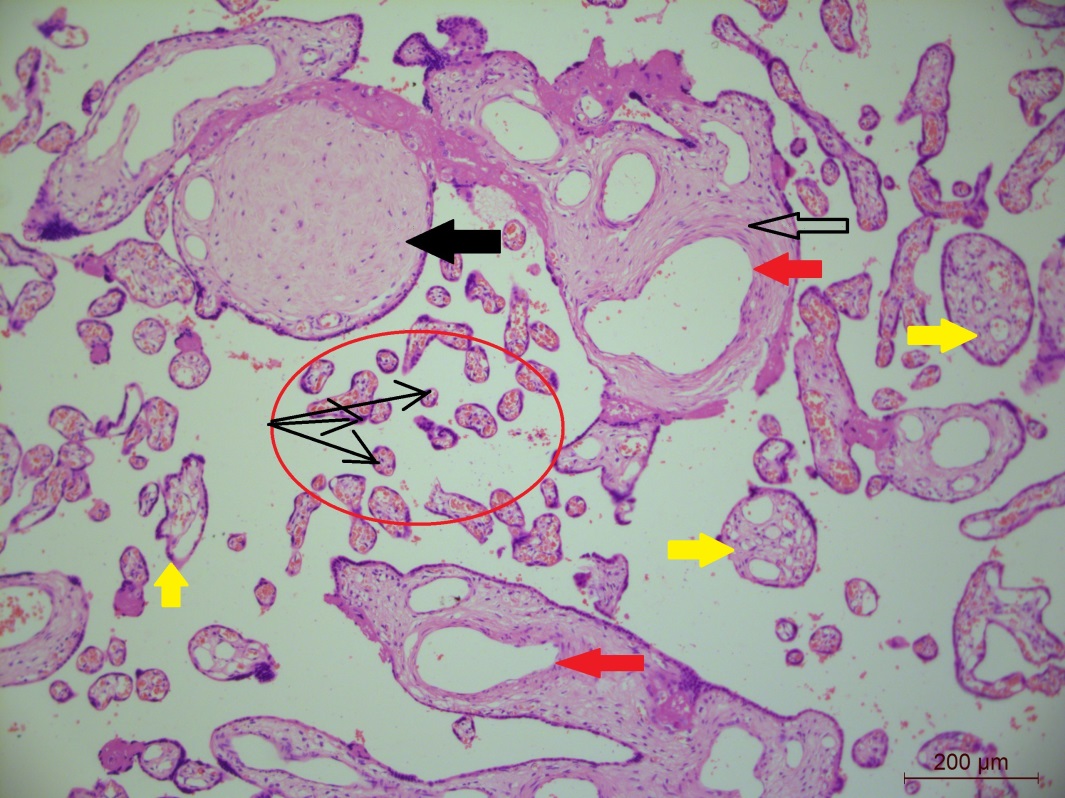


**Supporting Figures Captions**

**Fig S1:** Graph showing Spearman correlation performed between COVID-19 IgG Antibodies levels in COVID-19 +ve mother’s blood and COVID-19 +ve cord blood which showed a positive significant correlation (two-tailed *p* value <0.0001), an r-value of 0.42 at a 95% confidence interval of 0.26 to 0.56.

**Fig S2**: Microphotograph shows chorionic villi in placenta from COVID-19 positive mothers (2 villi marked by bold yellow arrows), lined by multinucleated syncytiotrophoblasts (bold black arrows) with their stroma (long black arrows) infiltrated with acute inflammatory cells (bold red arrows) damaging the blood vessels. Original magnification, ×20

**Fig S3**: Microphotograph shows multiple terminal chorionic villi in placenta from COVID-19 positive mothers lined with dilated fetal capillaries (long black arrows), and one stem villous (bold orange arrow) and intervillous hemorrhage (thin red arrow). Original magnification, ×10

**Fig S4**: Microphotograph shows multiple chorionic villi in placenta from COVID-19 positive mothers lined by multinucleated syncytiotrophoblasts (bold black arrows) and those within the black circle and marked in the bold black arrows are small and hyper mature indicating accelerated villous maturation. The adjacent villi marked with bold yellow arrows are normal sized villi.Original magnification, ×10

**Fig S5**: Microphotograph shows chorionic villi in placenta from COVID-19 positive mothers with multiple terminal villi (within the red circle and also marked with long thin black arrows) being smaller in comparison to adjacent normal sized villi (marked in bold yellow colour arrows), thus indicating accelerated maturation. Few villi show fibrosis in their stroma (large bold black arrow and bold black arrow not filled) and dilated blood vessels (bold red arrows). Original magnification, ×10
